# Antimicrobial efficacy of nano-particles for crop protection and sustainable agriculture

**DOI:** 10.1186/s11671-024-04059-9

**Published:** 2024-07-15

**Authors:** Pallavi Jali, Srinivas Acharya, Gyanranjan Mahalik

**Affiliations:** 1 Department of Botany, Utkal University, Vani Vihar, Bhubaneswar, Odisha India; 2 Department of Environmental Science, Government Autonomous College, Phulbani, Odisha India; 3 Department of Botany, School of Applied Sciences, Centurion University of Technology and Management, Jatani, Odisha India

**Keywords:** Crop protection, Nanoparticles, Plant pathogens, Sustainable agriculture

## Abstract

Plant diseases cause colossal crop loss worldwide and are the major yield constraining component in agriculture. Nanotechnology, which has the possible to revolutionize numerous fields of science, innovation, drug, and agriculture. Nanotechnology can be utilized for combating the plant infectious diseases and nano-materials can be utilized as transporter of dynamic elements of pesticides, host defense etc. to the pathogens. The analysis of diseases, finding of pathogens may turn out to be substantially more precise and fast with the utilization of nanosensors. As worldwide demand for food production raises against an evolving atmosphere, nanotechnology could reasonably alleviate numerous challenges in disease managing by diminishing chemical inputs and advancing quick recognition of pathogens. The major goal of this review is to increase growth and productivity using supplements with nanoparticles. (i.e., metals, metal oxides, and carbon) to treat crop diseases and make agricultural practices more productive and sustainable. Prominently, this improved crop may not only be straight connected to the diminished occurrence of pathogenic microorganisms, yet in might possibly add nutritional benefits of the nanoparticles themselves, particularly for the micronutrients important for generating host resistance.

## Introduction

Agriculture is the foundation of most nations and it gives food to people, legitimately and in a roundabout way. The global agricultural production is widely thought to need to expand or rise in order to take care of a rapidly developing total population, which is predicted to reach eight to nine billion by 2050. Production of agrifood is of indispensable significance, as it is one of the essential driving forces of economy. Due to the extreme pressure that environmental change, resource and energy shortages, and the rapidly expanding worldwide populace are placing on food and water resources, agricultural and horticultural practices are frequently in the news. Farming is getting progressively significant in the world of reducing resources and an ever-expanding worldwide populace [1, 2]. Given the expanding worldwide populace, it is important to utilize the advanced innovations.

Since materials' physical, biological, and chemical properties differ at this scale, the term "nanoscale" references to measurements that are typically made between 1 and 100 nm. "1 billionth of a metre" is referred to as a nanometer (nm). An application of nano-technology is the understanding and manipulation of matter at the nanoscale due to a special phenomenon [3]. "A nanomaterial is any type of material that has at least one (or more) dimensions at the nanoscale". Agriculture and related fields, such as hydroponics and fisheries, could be revolutionized by nanotechnology. Nano-agriculture involves utilizing nano-sized particles with remarkable properties to support crop growth, animals’ productivity and profitability [4, 5]. Application of nanotechnology to the horticulture, food divisions is recent contrasted to its utilization in drug delivery and drugs production [6] and it can possibly ensure protection of plants, screen plant development, distinguish plant diseases, enhance worldwide food production, and upgrade food quality [7–13]. Agriculture and food are the most significant fields of nanotechnology and its application [14–19].

Plant microbes cause critical decrease in crop productivity, with assessed worldwide colossal loss of 20–40% annually (Table 1) [20]. Currently management of pests depends intensely on the utilization of pesticides, for example, fungicides, insecticides and herbicides. Besides numerous benefits, like higher accessibility, and quick action, pesticides have several toxic effects towards non-target organisms, like generation of resistance and the resurgence of pest population [21]. Besides, it is assessed that approximately 90% of insecticides those are applied are lost during the procedure [21–23]. The utilization of nanoparticles to ensure plant protection can be through two distinct mechanisms: (a) nanoparticles themselves acting as crop protectant, or (b) nanoparticles as transporters or transporters for existing insecticides or dsRNA. It can be applied through spraying or imbibing the seeds, foliage and roots. Nanoparticles, as transporters or carriers, can give a few advantages, similar to (i) upgraded shelf life for usability, (ii) enhanced the solubility of pesticides that are poorly water soluble, and (iii) decreased toxicity [24]. This review emphasizes on the advanced methods of crop protection against pathogens using nano-particles and sustainable agriculture.

**Table 1:** List of some diseases caused by pathogenic microorganisms [30]

Crop	Pathogen	Disease
Rice	*Pyricularia oryzae*	Blast
	*Xanthomonas oryzae*	Bacterial blight
Wheat	*Puccinia recondiata*	Leasf rust
	*Puccinia striiformis*	Stripe rust
Maize	*Helminthocporium maydis*, *H. turcicum*	Leaf blight
Sorghum	*Sphacelotheca reiliara*	Grain mould
Pigeonpea	*Fusarium udum*	Wilt
Chickpea	*Fusarium oxysporum*	Wilt
Brassica	*Alternaria brassicaceae*	Blight
Soybean	*Phakospora packyrhizi*	Rust
Potato	*Phytopthora infestans*	Late blight
	*Streptomyces scabies*	Scab
	*Clavibacter michiganensis*	Ring rot
Citrus	*Xanthomonas citri*	Citrus canker
Carrot	*Erwinia caratovora*	Soft rot
	*Alternaria radicina*	Black rot
Cotton	*Xanthomonas malvacearum*	Angular leaf spot
Apple	*Erwinia amylovora*	Fire blight
Brinjal	*Pseudomonas solancearum*	Bacterial wilt
Pumpkin	*Pseudomonas syringae*	Angular leaf spot
Mango	*Xanthomonas campestris*	Bacterial canker
Banana	*Pseudomonas solancearum*	Moko disease
	*Erwinia caratovora*	Rhizome rot
Cauliflower	*Pseudomonas syringae* pv. *maculicola*	Bacterial leaf spot
Oats	*Xanthomonas campestris* pv. *translucens*	Bacterial streak

## Crop loss due to pathogens

Fungal infections cause significant agricultural losses. Although a wide range of fungicides is easily obtainable, improper application of these chemical fungicides may have negative effects on plant health (Table 1). A number of diseases, including anthracnose (*Glomerella lindemuthiana*), dry root rot (*Macrophomina phaseolina*), leaf blight (*Xanthomonas campestris* pv. vignicola), leaf crinkle disease (Urdbean leaf crinkle virus), leaf spot (*Cercospora canescens*), rust (*Uromyces appendiculatus*), and Mungbean yellow mosaic virus can all have a significant negative economic impact on the production of black gram [25]. Although the etiology and plant immunological mechanisms of maize white spot disease may be comparable to those of other maize leaf spot diseases that lower maize productivity, the disease is most likely a bacterial maize leaf spot caused by *Pantoea ananatis* [26]. At a projected expense of £0.12 million, the traditional biological control intervention of Papaya mealybug employing *Acerophagus papayae* benefited Ghanaian farmers and consumers to the tune of £1.1 million and £0.97 million over three years (2011–2013), respectively [27]. A natural, sustainable, and potentially effective therapy for gray mold is scopoletin, particularly when paired with triadimefon, which appears to block B. cinerea's HOG biological pathway [28]. Crop disease and pests might be controlled by the several paths that nanomaterials most likely function through. It is still challenging to identify the boundary between toxicity and advantageous benefits, and the resulting toxicity differed across the several cultivars under examination [29]. Nanotechnology might be a game-changer in the future when it comes to tackling this issue.

## Nanoparticles for crop protection and disease management

### Nanoparticles (NPs) as protectants against pathogenic microorganisms

Plant pathogenic microorganisms are significant restricting factors in the generation of food and agriculture [31, 32]. Various techniques are utilized to control microorganisms yet none of them offer ideal control of the diseases caused by the pathogens [33]. Subsequently, an incredible scope for the exploitation of nanotechnology in the field of crop protection and management of pathogenic plant microorganisms exists. Nanoparticles alone can possibly be straightforwardly applied to seeds, roots or foliage for resistance against pathogens and several pests (Table 2). Metal nanoparticles have been intensively explored for their antimicrobial properties [34–36]. Silver nanoparticles because of "green synthesis" have been highly popular [37]. Silver nanoparticles have indicated antifungal activity against several pathogenic fungal groups like *Alternaria alternata*, *Sclerotinia sclerotiorum*, *Macrophomina phaseolina*, *Rhizoctonia solani*, *Botrytis cinerea* and *Curvularia lunata* [38]. At the point, upon spray of silver nanoparticles over leaves of bean plants, total inhibition of infection was reported [39]. Zinc nanoparticles have been shown to have antibacterial action against *Pseudomonas aeruginosa*, with the greatest inhibition zone (221.8 mm) being observed at a concentration of 25 ng mL^−1^ ZnO NPs [40]. According to the reports, ZnO NPs are a unique antibacterial material. ZnO nanoparticles have been found to have fungicidal effects on *Penicillium expansum* and *Bortryis cinera*, two postharvest harmful fungi (Table 2). Ag NPs/PVP, a combination of polyvinylpyrrolidone and silver nanoparticles, have been thought to have antibacterial activity against gram positive and gram negative bacteria’s [41]. CuO NPs have been shown to be effective at inhibiting the growth of bacteria such *Staphylococcus aureus, Pseudomonas aeruginosa* and *Escherichia coli* [41]. High antibacterial and bactericidal power was shown by silver nanoparticles against methicillin-resistant gramme positive bacterial species [42].

**Table 2:** Nanoparticles as carrier for fungicides

Fungicide	Nanoparticle	Crop	Target Microorganism	References
Carbendazim	Chitosan or Pectin	*Cucumis sativus* *Zea mays*, *Solanum lycopersicum*	*Fusarium oxysporum &* *Aspergillus parasiticus*	[54]
Pyraclostrobin	Chitosan/MSN	–	*Puccinia asparagi*	[55]
Clove essential oil *Cymbopogon martini* essential oil	Chitosan	– *Zea mays*	*Aspergillus niger* *Fusarium graminearum*	[56, 57]
Tebuconazole Propineb Fludioxonil	Silver		*Bipolaris maydis*	[58]
Ferbam	Gold	Tea leaves	–	[59]
Prochloraz	PHSN	*Cucumis sativus*	*Botrytris cinerea*	[60]
Tebuconazole	Polymeric and SLN	Bean seeds		[61]
Azoxystrobin Difenoconazole	PBS/PLA	–	–	[62]
Tebuconazole Chlorothalonil Chlorothalonil	PVP and PVP copolymer	Southern pine sapwood Sothern yellow pine Birch and Suthern yellow pine	*Gloeophyllum trabeum,* *Tinea versicolor*; *G. trabeum*	[63–67]

Elbeshehy et al. [43] observed that bean plants exposed to a yellow mosaic infection and then scattered with Ag nanoparticles produced noticeably improved results (after 24 h of application of nanoparticles) than when they were applied prior to the appearance of the infection or simultaneously with inoculation of the pathogen. Ag nanoparticles demonstrated gigantic potential for management of plant disease however several difficulties related to them, for example, toxicity, soil interactions, and productivity [34, 44]. Poly-scattered gold nanoparticles through a mechanical grating supposedly melted and reduced the yellow mosaic of Barley providing protection against the virus [45]. The inherent features of chitosan nanoparticle are perfect, including lower toxicity to living things and "biodegradability, biocompatibility, non-allergenicity, and antimicrobial efficacy" [46]. It is reported to obstruct viral infection in plant tissues by securing them against diseases such as alfalfa mosaic virus, potato, peanut and cucumber viruses [47–49]. Chitosan nanoparticles have shown antibacterial capabilities, and they have been used to treat rice plants for *Pyricularia grisea* and *Fusarium crown*, as well as tomato root rot and Botrytis rot in grapes [30], but are less effective in combating microorganisms [50]. Chitosan may have antimicrobial effects in a variety of ways, including agglutination, H + / ATPase inhibition, cell membrane disruption, suppression of microbial growth and toxin generation, inhibition in mRNA and proteins, and obstruction of nutrient flow, according to Malerba and Cerana [51]. Chitosan showed antiviral impacts such as against *Bean mosaic* infection, *Tobacco mosaic* infection and *Tobacco necrosis* infection [51]. Copper sulphate (CuSo_4_) and Sodium tetraborate decahydrate (Na_2_B_4_O_7_) were discovered best in regulatory rust infections of peas. Micro-elements, for example, Mn and Zn likewise stifled the damping off and charcoal rot diseases in sunflower plants [52]. AG NPs/PVP were tried for fungicidal effects against *Candida* species.

### Nanoparticles serve as carriers

Nano-particles are utilised as carriers to entrap, encapsulate, absorb, or attach particles in order to effectively build agricultural and horticultural formulations (Table 2). Silica nanoparticles are excellent delivery vehicles because they may be carefully regulated for size, shape, and structure [53]. For example, mesoporous silica nanoparticles (MSNs) and porous hollow silica nanoparticles (PHSNs) have spherical shapes with pore-like holes in them. The shell structure of porous hollow silica nanoparticles secures the molecules inside the nanoparticles against destruction by ultraviolet light. Several studies recommend that Si has been utilized to upgrade plant resilience against different abiotic and biotic stresses and hence, Si nano-particles appear to be the characteristic strategy for the improvement of agriculture [68]. Chitosan nanoparticles have less dissolving ability in aqueous solutions, because of their hydrophobic nature [50, 69]. To boost its solubility, chitosan is therefore typically combined with an organic or inorganic copolymer [50]. It enhances the characteristics of chitosan because it contains reactive amine and hydroxyl groups [69, 70]. The advantages of solid lipid nanoparticles (SLNs) are that they provide a matrix to ensure the trapping of lipophilic compounds without the need of solvents (organic) and can cause regulated release of many components [71, 72]. Solid lipid nanoparticles are made of lipids, which are solid at ambient temperature. “Layered double hydroxides (LDHs) are clays that forms hexagonal sheets with layers of active molecules entrapped in the interlayer space” [73]. LDH nanoparticles degrade under acidic environments, for example, moisture and carbon dioxide from the air can breakdown LDH [74, 75].

### NPs to improve crop disease resistance

Because of their greater efficiency in controlling pests, reduced cost, and environmental friendliness, nanopesticides have emerged as a possible substitute for conventional pesticides [76]. Nanotechnology has great promise for controlling the concentration of secondary metabolites, generating genes that resist illness, distributing hormones and biomolecules, and producing transgenic plants that are more resistant to disease [77, 78]. According to Cao and Wang (2022) [79], NPs outperform ions, bulk particles, and commercial pesticides in the management of agricultural diseases by factors more than 1.5, 2.5, and 1.5, respectively. Mesoporous silica nanoparticles (NPs) therapies applied to the roots of watermelon reduced the disease severity by 40% and affected many genes linked to stress [80]. The antifungal and antibacterial properties of gold nanoparticles were demonstrated by Gautam et al. (2020) [81] against the fungus *Erwinia* sp., *Bacillus megaterium*, *Pseudomonas syringe*, *Fusarium graminearum*, *Fusarium avenaceum*, and *Fusarium culmorum*. When metsulfuron methyl-loaded polysaccharide nanoparticles (NPs) are applied topically to weeds growing in wheat, the weed biomass is significantly reduced in comparison to traditional herbicides [82]. Cu NPs were effective against five different bacterial species growth namely, *Agrobacterium tumefaciens*, *Dickeya dadantii*, *Erwinia amylovora*, *Pectobacterium carotovorum*, and *Pseudomonas savastanoi* [83].

## Nanotechnology in sustainable agriculture

### Role of nano-fertilizers on crop improvement

Nanofertilizer innovation is inventive and substituting strategies for its application is an approach to deliver nutrient supplements into the soil slowly and in a controlled manner, preventing Eutrofication and contamination of water resources [84, 85]. The development and productivity of horticultural crops have been seen to be enhanced by micronutrient nano-fertilizers, indicating encouraging outcomes. According to Ahmed et al. and Rivera-Gutierrez et al. [86, 87] these nano-fertilizers include trace elements including zinc, iron, boron, and copper, which are necessary for a number of physiological and metabolic activities in plants. Treatment with TiO_2_ nanoparticles had a considerable impact on maize development, whereas TiO_2_ treatment had little of an impact. Titanium nanoparticles improved photon energy transmission and light absorption. Another study discovered that the activity of the nitrate reductase enzyme in soybeans increased when a mixture of SiO and TiO nanoparticles was applied to the plant's leaves [88]. Urea-hydroxyapatite nanohybrids increase agronomic nitrogen usage efficiency in agricultural field testing by around 30% as compared to pure urea [89]. Furthermore, many measurements have shown that the high specific surface area and density of NPs in nanohybrids cause their high reactivity [90]. Nano fertilizers have exceptional highlights like high absorption, increased productivity, improved photosynthesis, and expansion of leaves' surface area [91]. When compared to control plants, pot cultures with foliar spraying showed that plants showered with 20 mg/mL zinc oxide nanoparticle showed enhanced growth and biomass output [92, 93]. In nano fertilizers, nutrient supplements can be encapsulated using nanomaterials and delivered as emulsions or nanoparticles [94]. The fertiliser or compost capsules' coating and binding of nano- and subnano-composites aid in controlling the release of nutrients [95]. To demonstrate how grain crops may more efficiently absorb and use additional resources, researchers used a nanocomposite of nitrogen, phosphorus, potassium, micronutrients, mannose, and amino acids [96]. Chemicals that influence plant growth have been released under control using zinc-aluminum layered double hydroxide nanocomposites. When used as a super fertiliser, carbon nanotubes have been shown to penetrate tomato seeds and influence their germination and growth rates. Science demonstrated that the dense seed coat may be pierced by carbon nanotubes, supporting water absorption into seeds [97, 98].

### Nanotechnology to enhance crop yields

Productivity has been demonstrated to increase when nanoparticle fertiliser is administered topically [99]. Many nanomaterials, mainly made of metal and carbon, have been examined in connection to their translocation, absorption, accumulation, and in particular their impacts on growth and development in a variety of agricultural plants [100, 101]. Examples of the advantageous morphological impacts include the increased germination rate, root and shoot length, and vegetative biomass of seedlings in many agricultural plants, including maize, wheat, ryegrass, soybean, tomato, radish, lettuce, spinach, onion, pumpkin, and cucumber. Improvements in a variety of physiological processes, such as enhanced photosynthetic activity and nitrogen uptake by metal-based nanomaterials in a select few crops, including soybean [102], spinach [103], and peanut [104], were noted. Nanobiotechnology imparts several technologies to improve agricultural quality and productivity through improvements in genetic level of plants and gene delivery and transport of drug molecules to site-specific cellular levels [105]. Nano-titanium dioxide, generational transmission of fullerol through rice seeds, and changes in gene expression at the cellular level caused by multiwalled carbon nanotubes in tomato and tobacco plants [106, 107] have all been used to support the hereditary implications of positive changes caused by nanoparticles. Because of the recovery reactions of the plant metabolic processes, it has been observed that germinating maize seeds subjected to magnetic fluids followed by exposure to electromagnetic field induce an articulated increase in nucleic acid [108, 109]. Tetramethylammonium hydroxide-coated magnetic nanoparticles caused maize's chlorophyll-a content to rise. Additionally, iron oxide use in pumpkin was found to encourage root elongation by Wang et al. [110]. Positive effects on strawberry development and production have been shown when zinc oxide (ZnO) and iron oxide (FeO) nanoparticles are added at a concentration of 150 parts per million (ppm) each [111]. Mesoporous silica nanoparticle therapies applied to the roots of watermelon reduced the disease severity by 40% and affected many genes linked to stress [80]. It has been demonstrated that FeO-NPs not only promote plant development but also lower the Na + and Cl- concentration in wheat grains [112]. Mahmood and Hussain reported After wasted tea was gasified catalytically, 60% liquid extract, 28% fuel gases, and 12% charcoal were produced. 53.03% ethene, 37.18% methanol, and 4.59% methane are found in gaseous products [113]. A nanotechnology-based approach might complement an environmentally friendly agricultural production method [114].

## Conclusion and future perspectives

The possible usages and advantages of nano-technology are tremendous. It is alluring to encourage agricultural practices, boost output while lowering inputs through better monitoring and focused action, and increase yield efficiency with nanotechnology. Because of nanotechnology, plants can utilise water, pesticides, and fertilizers more effectively. The use of nanotechnology may benefit both the agri-food industry through the development of cutting-edge goods and farmers through increased food output. The application of nanoparticles in crop protection and production has the potential to revolutionize the way we grow and harvest crops, offering sustainable solutions to meet the rising demand for high-quality and nutritious food. However, to achieve these goals and take advantage of new opportunities, future research should concentrate on optimizing nanoparticle formulations, improving their delivery methods, and evaluating their long-term effects on plant growth, fruit quality, and the environment. To pinpoint the limitations, minimize the detrimental effects, and optimize the advantages, more study is required. The capacity of nanotechnology to function within the confines of a sustainable agricultural system will ultimately determine its fate. NPs can exhibit antibacterial capabilities either by themselves or in conjunction with antibiotics, which helps to mitigate the current problem of acquired resistance brought on by overuse or abuse of antibiotics. Future research should concentrate on two areas in light of this possible application: evaluating the safety aspects of NP use and reducing the environmental effect of NP synthesis.

## Data Availability

Not applicable.
